# Comprehensive Analysis of the Glycolysis-Related Gene Prognostic Signature and Immune Infiltration in Endometrial Cancer

**DOI:** 10.3389/fcell.2021.797826

**Published:** 2022-02-11

**Authors:** Xiao Yang, Xingchen Li, Yuan Cheng, Jingyi Zhou, Boqiang Shen, Lijun Zhao, Jianliu Wang

**Affiliations:** Department of Obstetrics and Gynecology, Peking University People’s Hospital, Beijing, China

**Keywords:** endometrial cancer, glycolysis, prognostic signature, WGCNA, bioinformatics, immune infiltration

## Abstract

Glucose metabolic reprogramming and immune imbalance play important roles in the progression of cancers. The purpose of this study is to develop a glycolysis-related prognostic signature for endometrial cancer (EC) and analyze its relationship with immune function. The mRNA expression profiling of the glycolysis-related genes and clinical data of EC patients were downloaded from The Cancer Genome Atlas (TCGA). We identified a glycolysis-related gene prognostic signature for predicting the prognosis of EC by using The Least Absolute Shrinkage and Selection Operator (LASSO) regression and found the patients in the high-risk group had worse survival prognosis. Multivariate Cox regression analysis showed that the gene signature was an independent prognostic factor for EC. The ROC curve confirmed the accuracy of the prognostic signature (AUC = 0.730). Then, we constructed a nomogram to predict the 1–5 years survival rate of EC patients. The association between the gene signature and immune function was analyzed based on the “ESTIMATE” and “CIBERSORT” algorithm, which showed the immune and ESTIMATE scores of patients in the high-risk group were lower, while the low immune and ESTIMATE scores were associated with a worse prognosis of patients. The imbalance of immune cells was also found in the high-risk group. Further, the protein of CDK1, a gene in the signature, was found to be closely related to prognosis of EC and inhibition of CDK1 could inhibit migration and promote apoptosis of EC cells. This study reveals a link between glycolysis-related gene signature and immunity, and provides personalized therapeutic targets for EC.

## Introduction

Endometrial cancer (EC) is one of the most common gynecological malignancies. The latest cancer statistics of the American Cancer Society showed that the number of new cases in the United States increased by 63,230, with 11,350 deaths being reported, and the incidence rate ranked fourth in female malignant tumors and sixth in deaths in 2018 (Sheikh et al., 2014; Siegel et al., 2018). In addition, the 5-years disease-free survival rate and 5-years overall survival rate of patients with EC were 82.3 and 81%, respectively, and the tumor recurrence rate and tumor-related mortality rate were 14.5 and 15.9%, respectively (Tejerizo-García et al., 2013). Although early diagnosis, surgery, radiotherapy, and chemotherapy can significantly improve the survival times of patients, the treatment for early patients with the need for fertility preservation, advanced tumor, and relapse is still limited. Therefore, it is urgent to explore new prognostic biomarkers and therapeutic targets.

Metabolic reprogramming is one of the most important characteristics of tumor cells. Approximately 80% of glucose was used to produce ATP in tumor cells through aerobic glycolysis accompanied by lactic acid production even under aerobic environment, known as the “Warburg effect” (Hanahan and Weinberg, 2011). Studies have shown that the glucose metabolism reprogramming of tumor cells is closely related to the occurrence, progression, and chemotherapy resistance of tumors (Icard et al., 2018). It was reported that multiple genes could promote the progression of EC by promoting glycolysis (Han et al., 2019). Since glucose metabolism reprogramming is an important feature that distinguishes tumor cells from normal cells, it may be of great significance to explore prognostic genes and potential therapeutic targets of EC from the perspective of abnormal glucose metabolism.

Immune cells and stromal cells are two primary types of nontumor components in the tumor microenvironment and have been proposed to be of considerable importance for tumor diagnosis and prognosis evaluation (Ren et al., 2018). Estimation of stromal and immune cells in malignant tumors using expression data (ESTIMATE) could predict tumor purity by analyzing gene expression (Yoshihara et al., 2013). Recently, a study has reported the relationship between the immune microenvironment and prognosis of patients with colorectal cancer metastasis, and found that the metastasis with the smallest number of immune cells entering represented the worst immune microenvironment; therefore, tumor immune escape was most likely to occur (Van den Eynde et al., 2018). Therefore, the infiltration of immune cells in tumors is closely related to the clinical outcome of patients.

Many studies have reported the relationship between glycolysis and tumor immunity (Justus et al., 2015; Cascone et al., 2018; Deng et al., 2018). It has been reported that enhanced glycolysis of tumor cells could become the main obstacle of targeted treatment of tumor immune cells by affecting the infiltration of immune cells in the tumor microenvironment, while interference with glycolysis of tumor cells could enhance the effective infiltration of antitumor immune cells (Ganapathy-Kanniappan, 2017). It is suggested that further study of the relationship between glycolysis and tumor immunity is of strong significance for the effective targeted treatment of tumors. However, studies investigating glycolysis genes and their prognostic value and relationship with immune function in patients with EC are limited. In this study, we analyzed the mRNA expression profiling of EC from The Cancer Genome Atlas (TCGA) and established a 10 glycolysis related gene signature by using LASSO regression analysis. Further, we constructed a nomogram based on the gene signature and clinicopathological factors to predict the prognosis of EC patients. In addition, we analyzed the immune scores and immune cell infiltration related to the glycolysis-related gene signature. Finally, CDK1, a glycolysis related gene in the signature was proposed to be related to the prognosis of EC and its function was validated *in vitro*.

## Materials and Methods

### Data Collection and Preparation

We downloaded mRNA expression profiling (FPKM format) of EC from the TCGA database, including 552 EC and 35 normal samples (https://portal.gdc.cancer.gov/). The corresponding clinicopathological information, including age, tumor stage, grade, metastasis, lymph node metastases, survival time, and survival status, were downloaded from the TCGA data portal.

### Screening Glycolysis Related Genes and Functional Enrichment Analysis

We downloaded the gene sets related to glycolysis from the Molecular Signatures database (MSigDB) of the Gene Set Enrichment Analysis (GSEA) website, including HALLMARK_GLYCOLYSIS, KEGG_GLYCOLYSIS_GLUCONEOGENESIS and REACTOME_GLYCOLYSIS (http://software.broadinstitute.org/gsea/index.jsp). Perl script was used to extract the expression matrix of glycolysis-related genes. The R “limma” package was used to screen the differentially expressed genes (DEGs), and the screening conditions were |logFC|>0.5 and false discovery rate (FDR) < 0.05. Volcano plots and heatmap clustering were conducted using R software. Gene ontology (GO) and Kyoto Encyclopedia of Genes and Genomes (KEGG) analyses were performed using the R “ClusterProfiler” package (Yu et al., 2012).

### Construction of the Prognostic Glycolysis-Related Gene Signature

A univariate Cox regression was used to screen the prognostic glycolysis-related DEGs, and *p* < 0.01 was considered to be statistically significant. LASSO regression was applied to establish the prognostic gene signature (Zhou et al., 2018). The risk score of the gene signature = (coef 1×expression of gene 1)+(coef 2×expression of gene 2)+.+(coef n×expression of gene n). Based on the risk score, the patients were divided into high- and low-risk subgroups for subsequent study. The overall survival of patients in the high- and low-risk subgroups were analyzed by using the R “survival” and “survminer” packages, and Kaplan-Meier (K-M) survival curves were drawn. The risk curve and survival state diagram were drawn by the R software package. Univariate and multivariate Cox regression analyses were used to analyze the prognostic factors of EC, and receiver operating characteristic (ROC) curves were drawn by the R “survivalROC” package. The nomogram was constructed using R software to integrate multiple prediction indicators based on multivariate Cox regression analyses (Tang et al., 2016).

### Estimation of Immune and Stromal Scores Related to Gene Signature

We used the “ESTIMATE” algorithm to calculate the immune scores (which capture the presence of stroma in tumor tissue), stroma scores (which capture the infiltration of immune cells in tumor tissue), and estimate scores (which infer tumor purity). According to the immune scores, stroma scores, and ESTIMATE scores, the patients were divided into high- and low-score subgroups by using the median value as the threshold. The overall survival of patients in the low- and high-score subgroups was analyzed by the R “survival” package. The immune scores and stromal scores of patients in the high-risk and low-risk subgroups were also calculated by ESTIMATE, and the immune scores and stromal scores of the high-risk subgroup and low-risk subgroup were compared by the Wilcoxon test; *p*-values < 0.05 were considered to be significant.

### Association Between the Gene Signature and Tumor-Infiltrating Immune Cells

The mRNA expression profiling of EC was transformed into a matrix of immune cells based on CIBERSORT software. The difference in tumor-infiltrating immune cells between the high-risk subgroup and low-risk subgroup was screened by the R “limma” package, and the screening condition was *p* < 0.05. The cor. test in R was used to analyze the correlation coefficient between the 10 glycolysis-related genes and immune cell infiltration, and the correlation graph was drawn by the R “ggcorrplot” package.

### A Gene Co-expression Network Was Built by the WGCNA

We conducted the weighted correlation network analysis (WGCNA) to identify the potential mechanisms associated with the gene prognosis model. We first filtered out the best soft threshold by “WGCNA” R package to maintain sufficient connectivity and keep the gene network close to the scale-free topology. Second, we performed the analysis module associated with the risk model and other clinical factors. Furthermore, the key genes were identified from the WGCNA analysis. Then, the metascape online website (https://metascape.org/gp/index.html) was used to perform the GO and KEGG analysis about the key genes related with the gene prognosis model (*p* value cutoff: 0.01). Also, GSEA software was used to analyze the significantly enriched signal pathways between high-risk and low-risk groups (using FDR<0.05 as the cut-off criterion).

### Genetic Alteration, Co-expression, and Neighbor Gene Network Analyses

The cBioPortal website (https://www.cbioportal.org/) developed by Memorial Sloan Kettering Cancer Center (MSKCC) is a comprehensive open network platform based on the TCGA database that integrates data mining, data integration, and visualization (Gao et al., 2013). The genetic alterations of 10 glycolysis-related genes were obtained from cBioPortal based on TCGA. There were 548 EC samples (TCGA, Firehose Legacy) analyzed. Mutations and mRNA expression z-scores (RNA Seq V2 RSEM) with a z-score threshold ±2 were selected. The protein-protein interactions (PPI) network was constructed by using the STRING database (http://string-db.org/) to screen the proteins that have the closest relationship with the 10 genes with high confidence 0.700. IntAct database was also used to construct a network including the physical association and direct interaction between the 10 genes and related proteins (https://www.ebi.ac.uk/intact/). A protein regulatory network of CDK1 was constructed based on the BioGRID database (https://thebiogrid.org/). The expression of glycolysis-related genes in the gene signature was further validated at the protein level (The Human Protein Atlas database: http://www.proteinatlas.org). The clinical prognosis analysis of proteins corresponding to the genes in the signature was performed on the cancer proteome atlas (https://www.tcpaportal.org/tcpa/survival_analysis.html).

### External Validation Based on the Clinical Samples

The glycolysis-related gene signature was further validated by our own clinical data including 24 EC samples from surgical patients in the Department of Obstetrics and Gynecology, Peking University People’s Hospital. Total RNA isolation and RNA sequencing were performed as previously reported (Yin et al., 2019). The patients were followed-up by February 2018. This study was approved by the Institutional Ethics Committee (Human Research) of Peking University People’s Hospital and informed consent was obtained from the patients.

### *In vitro* Validation

EC cell line Ishikawa was obtained from a gynecologic laboratory in Peking University People’s Hospital. The Ishikawa cells were cultured with DMEM/F-12 medium (Macgene, Beijing) containing 10% FBS in 5% CO_2_ incubator at 37°C.

Ro 3306 (MCE, HY-12529), a selective inhibitor of CDK1 was used to verify the function of CDK1 in EC cells. Ishikawa cells were inoculated into 96 well plates (3000 cells/well), and cell counting Kit-8 (CCK-8) was used to detect the effect of Ro 3306 on the proliferation of Ishikawa according to the instructions. The half maximal inhibitory concentration (IC50) was calculated.

To study the effect of Ro 3306 on the migration ability of endometrial cancer cells, scratch test and transwell were performed. Ishikawa cells were inoculated into six well plates. When the fusion degree of cells reaches 90%, a scratch was made by 100 μL tips. Ishikawa cells were treated with Ro 3306 5 μM, 10 μM, respectively and established the control group. The scratches were imaged at 0 and 48 h. For the transwell experiment, 8*10^4^ cells were inoculated into the upper chamber with serum free medium, and 500 μL medium containing 10% FBS was added in the lower chamber. After 36 h, the cells near the lower chamber were fixed by paraformaldehyde and stained with crystal violet. The invasion cells were photographed. Further, we performed flow cytometry to detect the effect of Ro 3306 on the apoptosis of Ishikawa. The cells were treated as above. After 24 h, the cells (5*10^5^) were harvested and incubated with 5 μL PI and 5ul Annexin-FITC (BD, 556547) for 20 min, then analyzed on flow cytometry.

## Results

### Glycolysis-Related Gene Sets Differ Significantly Between EC and Normal Samples

**FIGURE 1:**
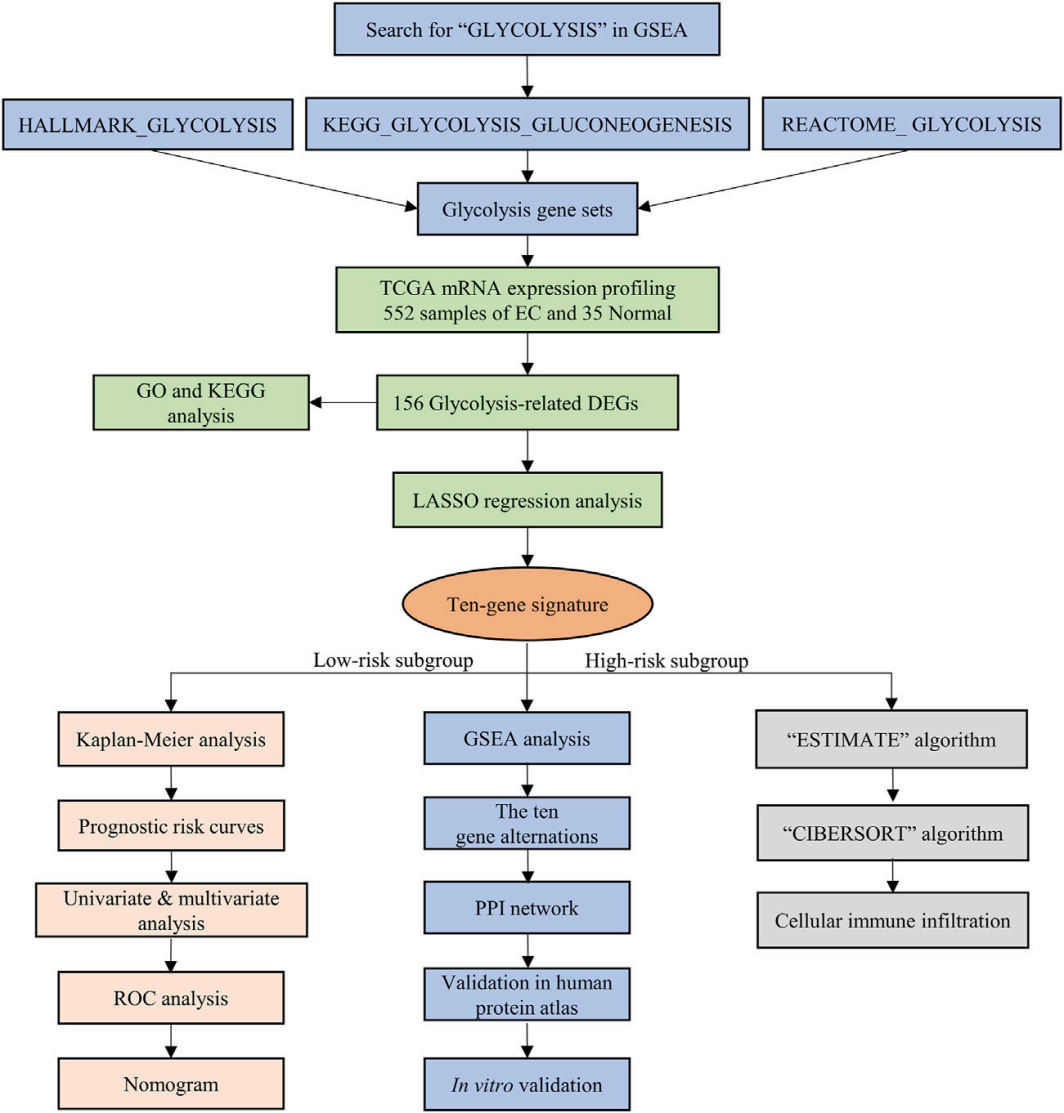
The flow chart of the study design and analysis.

**FIGURE 2:**
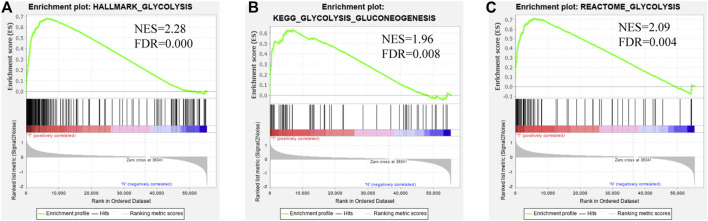
Enrichment plots of three glycolysis-related gene sets which were significantly differentiated between normal and EC tissues using GSEA. (FDR is the corrected *p* value of the multiple hypothesis test, NES stands for normalized enrichment score) **(A)**. Glycolysis-related HALLMARK gene sets **(B)**. Glycolysis-related KEGG gene sets **(C)**. Glycolysis-related REACTOME gene sets.

The mRNA expression profiling of EC was downloaded from TCGA, including 35 normal and 552 EC samples, and these data were analyzed as in the flowchart (Figure 1). GSEA software was used to analyze the enrichment of three glycolysis gene sets in the EC and normal groups. We found that there was a significant difference (FDR<0.01) in the three glycolysis-related gene sets between the EC group and the normal control group (Figure 2A–C).

### Identification of Glycolysis-Related DEGs

**FIGURE 3:**
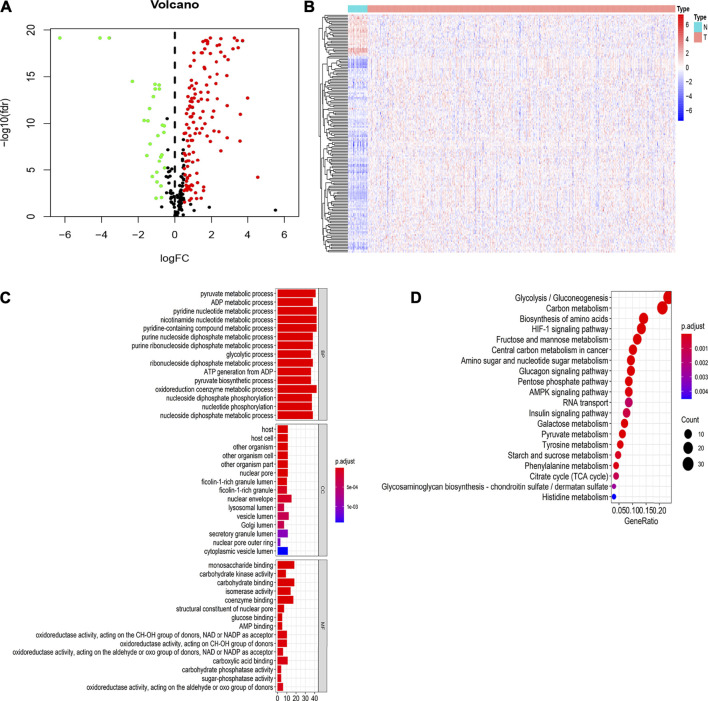
Identification of differentially expressed genes (DEGs) related to glycolysis of TCGA datasets between normal and EC tissues and GO and KEGG pathway enrichment analysis of DEGs **(A)**. Differential expression genes between two groups. The red dot is the up-regulated gene, the green dot is the down-regulated gene, and the black dot is the other genes without significant difference screened by the criteria of |Fold Change|>0.5 and FDR<0.05 **(B)**. Hierarchical clustering of differentially expressed genes in two groups **(C)**. The GO functional enrichment analysis of differential genes includes three domains: molecular function, biological process, and cell composition **(D)**. KEGG pathway analysis of differentially expressed genes.

Perl was used to extract the expression matrix of the selected glycolysis genes from mRNA expression profiling of EC. We further screened the differentially expressed glycolysis genes between the EC and normal group using the R “limma” package (FDR<0.05, |logFC|>0.5). The results showed that there were 156 DEGs, 128 of which were upregulated, and 28 of which were downregulated (Figure 3A). The R “heatmap” package was used to draw the heatmaps (Figure 3B).

To further verify whether these DEGs are related to glycolysis, we used the R “ClusterProfiler” package to analyze GO and KEGG enrichment. The results showed that in the biological process (BP), the DEGs were mainly involved in pyruvate metabolic process, the glycolytic process, and the oxidoreduction coenzyme metabolic process. In molecular function (MF), the DEGs are primarily involved in glucose binding, carbohydrate kinase activity, and sugar phosphatase activity. In the cell components (CC), the DEGs are mainly involved in the nuclear envelope and secreted granule lumen (Figure 3C). The results of KEGG enrichment analysis showed that the DEGs were mainly involved in glycolysis/gluconeogenesis, carbon metabolism, and the HIF-1 signaling pathway (Figure 3D). These results suggested that these DEGs were related to glycolysis in EC.

### Construction of the Glycolysis-Related Gene Signature to Predict Patient Outcomes

**FIGURE 4:**
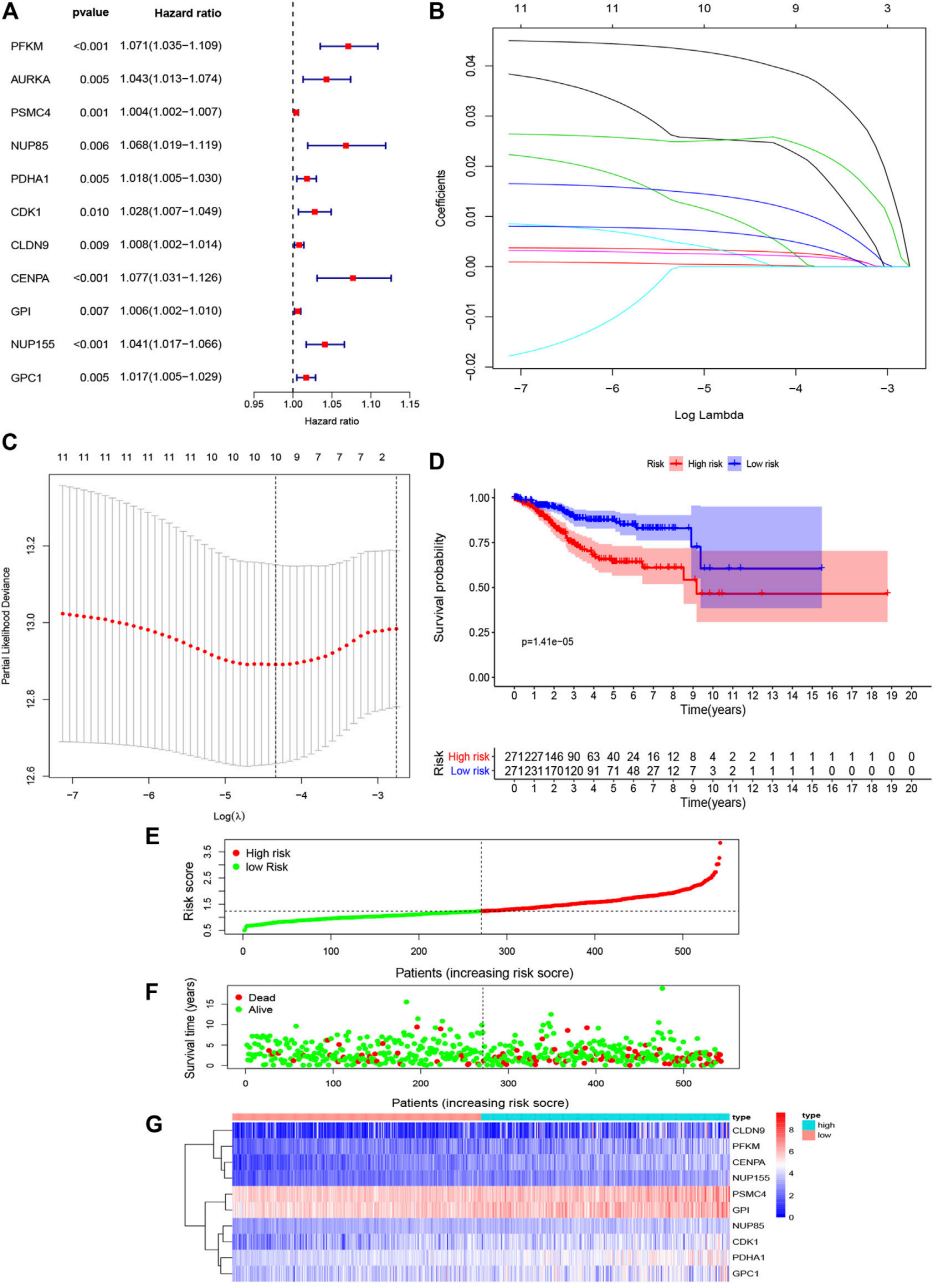
Construction and validation of prognostic model prognosis-associated genes of EC **(A)**. Results of univariate Cox analysis for the prognostic genes in EC. **(B,C)**. LASSO regression model **(D)**. Kaplan-Meier analysis for the 10 glycolysis-related gene signatures related to risk score predicts overall survival in patients with EC **(E)**. Gene signature-related risk score distribution in each patient **(F)**. Survival days of patients in order of the value of risk scores **(G)**. A heatmap of 10 glycolysis-related gene signatures.

We performed univariate Cox regression analysis and a total of 11 glycolysis related genes were screened to be closely related to the survival of EC patients. HR > 1 represents a risk gene (*p* < 0.05) (Figure 4A). Furthermore, the prognostic gene signature was constructed by LASSO regression analysis, and finally 10 genes were screened to establish the prognostic gene signature, namely, PFKM, PSMC4, NUP85, PDHA1, CDK1, CLDN9, CENPA, GPI, NUP155, and GPC1 (Figures 4B,C and Supplementary Table S1). The risk score of the gene signature= (0.0420×expression of PFKM) + (0.0032×expression of PSMC4)+(0.0097×expression of NUP85) + (0.0138×expression of PDHA1) + (0.0028×expression of CDK1) + (0.0024×expression of CLDN9) + (0.0252×expression of CENPA) + (0.0004×expression of GPI) + (0.0253×expression of NUP155) + (0.0067×expression of GPC1).

Based on this gene signature, all patients were divided into high- and low-risk subgroups using the risk score median as the threshold. K-M analysis showed that the overall survival rate of patients in the high-risk subgroup was significantly lower than that of the low-risk subgroup (*p* < 0.05) (Figure 4D). Then, patients were ranked according to the risk score, and the 10 gene signature were ranked according to the order of increasing risk score. The results indicated that the number of deaths increased with increasing risk score, and the expression levels of PFKM, PSMC4, NUP85, PDHA1, CDK1, CLDN9, CENPA, GPI, NUP155, and GPC1 were positively correlated with the risk score, which further confirmed that PFKM, PSMC4, NUP85, PDHA1, CDK1, CLDN9, CENPA, GPI, NUP155, and GPC1 were risk genes (Figure 4E–G).

**FIGURE 5:**
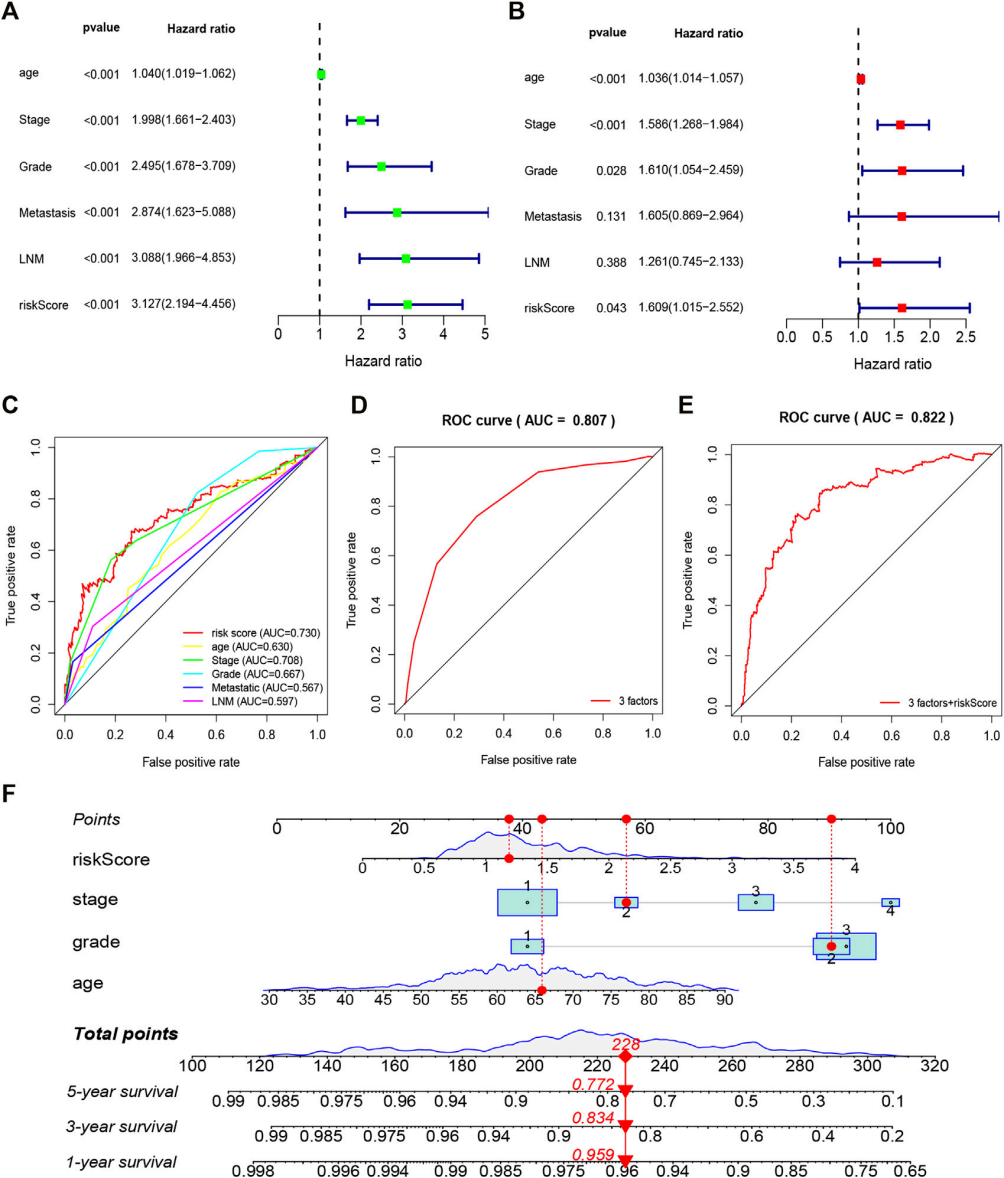
Forrest plot of the univariate and multivariate Cox regression analysis in EC **(A)**. The regression analysis of overall survival in EC **(B)**. The multivariate Cox regression analysis in EC **(C)**. ROC curve analysis was performed to evaluate the diagnostic efficacy of the gene signature and other clinical characteristics **(D,E)**. ROC curve analysis was performed to evaluate the diagnostic efficacy of the three factors (age, grade, and stage) and three factors + riskScore **(F)**. Nomogram is used to show the relationship between the variables in the prediction model and predict the 1–5 years overall survival rate of patients.

To further evaluate whether the constructed 10 gene signature is an independent prognostic factor for EC, we conducted univariate and multivariate Cox regression analysis (Figure5A,B). The results showed that risk score was an independent prognostic factor of EC, and age, grade, and stage were also independent prognostic factors (*p <* 0.05). To evaluate the clinical diagnostic ability of the 10 gene signature, we conducted ROC analysis. The results showed the risk score (area under the curve, AUC = 0.730), stage (AUC = 0.708), grade (AUC = 0.667), age (AUC = 0.630), LNM (AUC = 0.597), and metastasis (AUC = 0.567) (Figure 5C). In addition, the AUC of the survival assessment was 0.807 of three factors (age, grade, and stage) and 0.822 of three factors + riskScore (Figure 5D,E). The nomogram was also built based on the glycolytic gene signature and clinicopathological prognostic factors in EC (Figure 5F). These results suggested that the 10 gene signature has great potential significance in predicting EC prognosis.

### Estimation of Immune and Stromal Scores Related to Gene Signature

**FIGURE 6:**
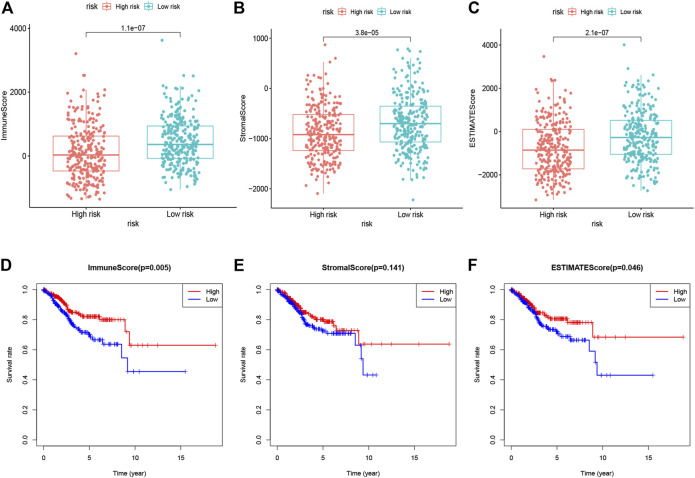
Immune scores, stromal scores, and ESTIMATE scores of 10 glycolysis-related gene signatures were related to risk score **(A)**. Immune scores for patients in the low- vs. high-risk group **(B)**. The stromal scores for patients in the low- vs. high-risk group **(C)**. The ESTIMATE scores for patients in the low- vs. high-risk group **(D)**. Kaplan-Meier analysis of overall survival for patients with low vs. high immune scores **(E)**. Kaplan-Meier analysis of overall survival for patients with low vs. high stromal scores **(F)**. Kaplan-Meier analysis of overall survival for patients with low vs. high ESTIMATE scores.

Since immune cells and stromal cells are two main types of nontumor components in the tumor microenvironment, they have been proposed to be valuable for tumor diagnosis and prognosis evaluation. Therefore, to further reveal the relationship between the tumor microenvironment and the 10 gene signature, we first estimated immune scores, stromal scores, and ESTIMATE scores of 552 EC samples in TCGA by the “ESTIMATE” algorithm. Immune scores and stromal scores are used to reflect the presence of immune cells and stromal cells, and ESTIMATE scores to represent the purity of the tumor. Further, we found that the immune scores, stromal scores, and ESTIMATE scores of the high-risk subgroup were lower than those of the low-risk subgroup (Figures 6A–C). More importantly, the overall survival rate of patients with low immune scores was significantly lower than that of patients with high immune scores, and there was no significant difference in the overall survival rate of patients with low and high stroma scores, while the overall survival rate of patients with low ESTIMATE scores was also lower than that of patients with high ESTIMATE scores (Figures 6D–F). These results indicated that the poor prognosis of patients in the high-risk group may be closely related to the lower immune cells and lower purity of the tumor.

### Correlation Between Immune Cell Infiltration and the 10 Gene Signature

To further verify the relationship between immune cell infiltration and the gene signature, we analyzed the proportion of 22 kinds of immune cells by using the “deconvolution method” of CIBERSORT software, and the samples were screened by *p* < 0.05. The results showed that compared with the low-risk subgroup, immune cells, such as activated dendritic cells, M1 macrophages, M2 macrophages, activated T memory cells, and T follicular helper cells, increased significantly in the high-risk group, while dendritic cell resetting, T cell memory resetting, and T regulatory (Treg) cells decreased significantly in the high-risk group (Supplementary Figure S1A). Furthermore, K-M analysis was used to screen the immune cells closely related to the prognosis of patients. The results indicated that the overall survival rate of patients in the high-proportion group of resting dendritic cells, activated NK cells, and regulatory T cells (Tregs) was significantly higher than that of the low-proportion group (Supplementary Figure S1B). Taken together, the poor prognosis of high-risk patients may be related to the imbalance of immune cells.

### WGCNA Analysis and Related Signaling Pathways of the Gene Signature

**FIGURE 7:**
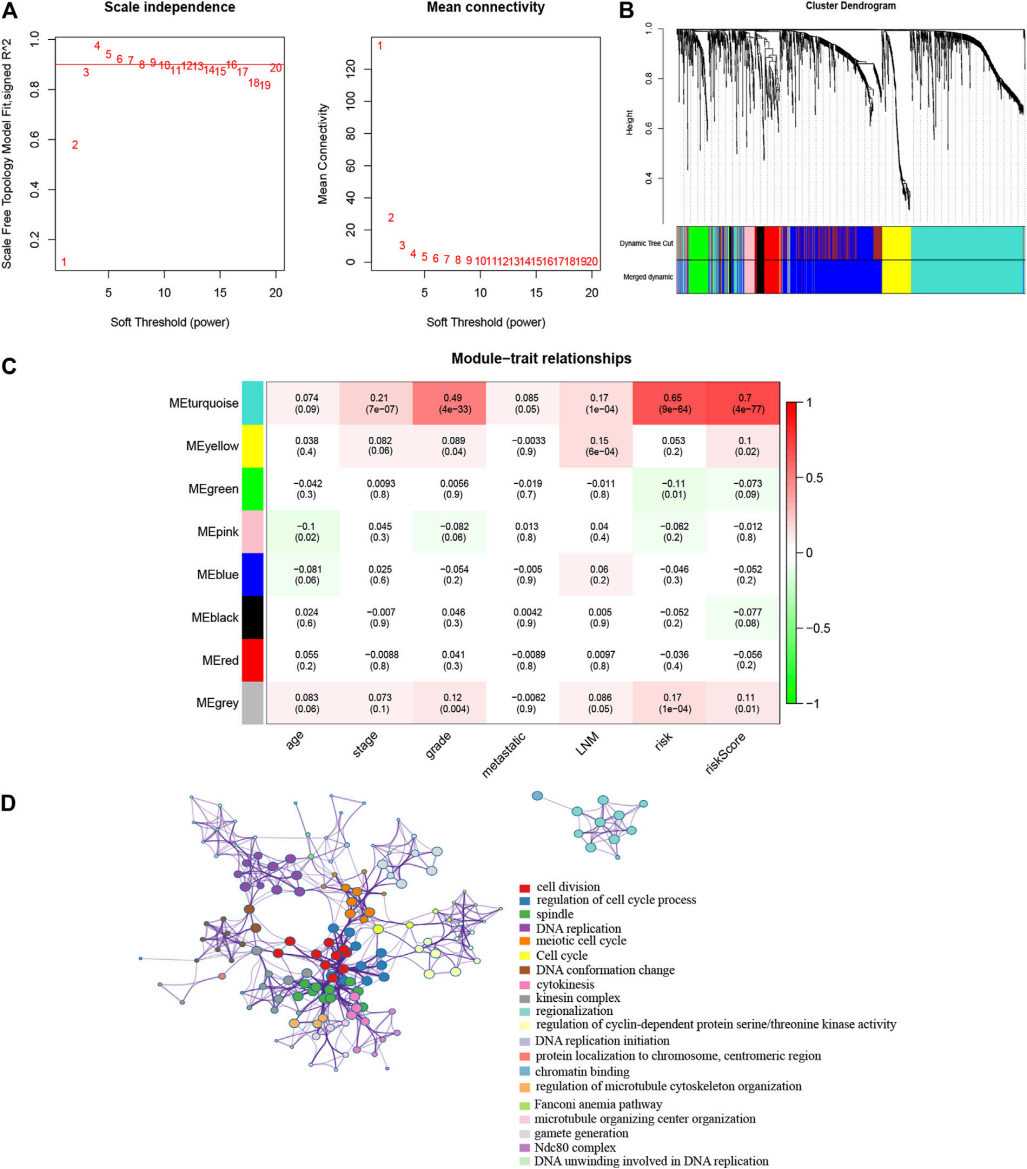
WGCNA was performed to identify the potential mechanisms associated with the prognostic signature **(A)**. Screening of the soft threshold **(B)**. Clustering dendrogram of genes in EC tissues **(C)**. Correlation analysis of gene modules with risk model and clinical traits **(D)**. Enrichment clustering network analysis in the Metascape database.

To better understand the network interaction between the risk model and other genes, we extracted mRNA expression profiling and clinical information for WGCNA analysis. The DEGs between cancer and normal samples were chosen. For constructing a weighted gene network, the threshold of the adjacency matrix should meet the criterion that the network is close to scale-free, and three was selected as the threshold for network construction (Figure 7A). These co-expression modules were then constructed, and the similar modules were clustered, and finally eight gene modules were obtained (Figure 7B). The results of correlation analysis of the gene modules with gene-signature and clinical traits showed that the turquoise module had the highest correlation with the gene-signature (Cor = 0.65, p = 9e-64 for risk; Cor = 0.7, p = 4e-77 for risk score) and the grade (Cor = 0.49, p = 4e-33) (Figure 7C). The turquoise module contained 604 genes, and we then analyzed these genes with Metascape. The GO and KEGG analyses were performed, and the top 20 clusters were chosen to construct a gene function clustering network (Figure 7D). These results indicated that the 10 gene signature was significantly associated with a gene module, which was also related with the clinical grade of patients. The gene module mainly enriched in cell division, regulation of cell cycle process and DNA replication, that were important biological processes in the tumor progression.

GSEA was used to analyze the enrichment signal pathways in the high-risk subgroup and low-risk subgroup. A total of 70 significantly enriched KEGG signaling pathways were screened (FDR<0.05). Many of these pathways are closely related to metabolism, including pyruvate metabolism, glycolysis gluconeogenesis, and inositol phosphate metabolism. Additionally, some signaling pathways are closely related to the occurrence and development of tumors, such as the cell cycle, EC, the ERBB signaling pathway, the MAPK signaling pathway, the mTOR signaling pathway, and the Wnt signaling pathway (Supplementary Figure S2). These results reveal the potential mechanism of the glycolytic prognosis model involved in EC.

### Comprehensive Analysis of Glycolysis Related Genes in the Gene Signature

**FIGURE 8:**
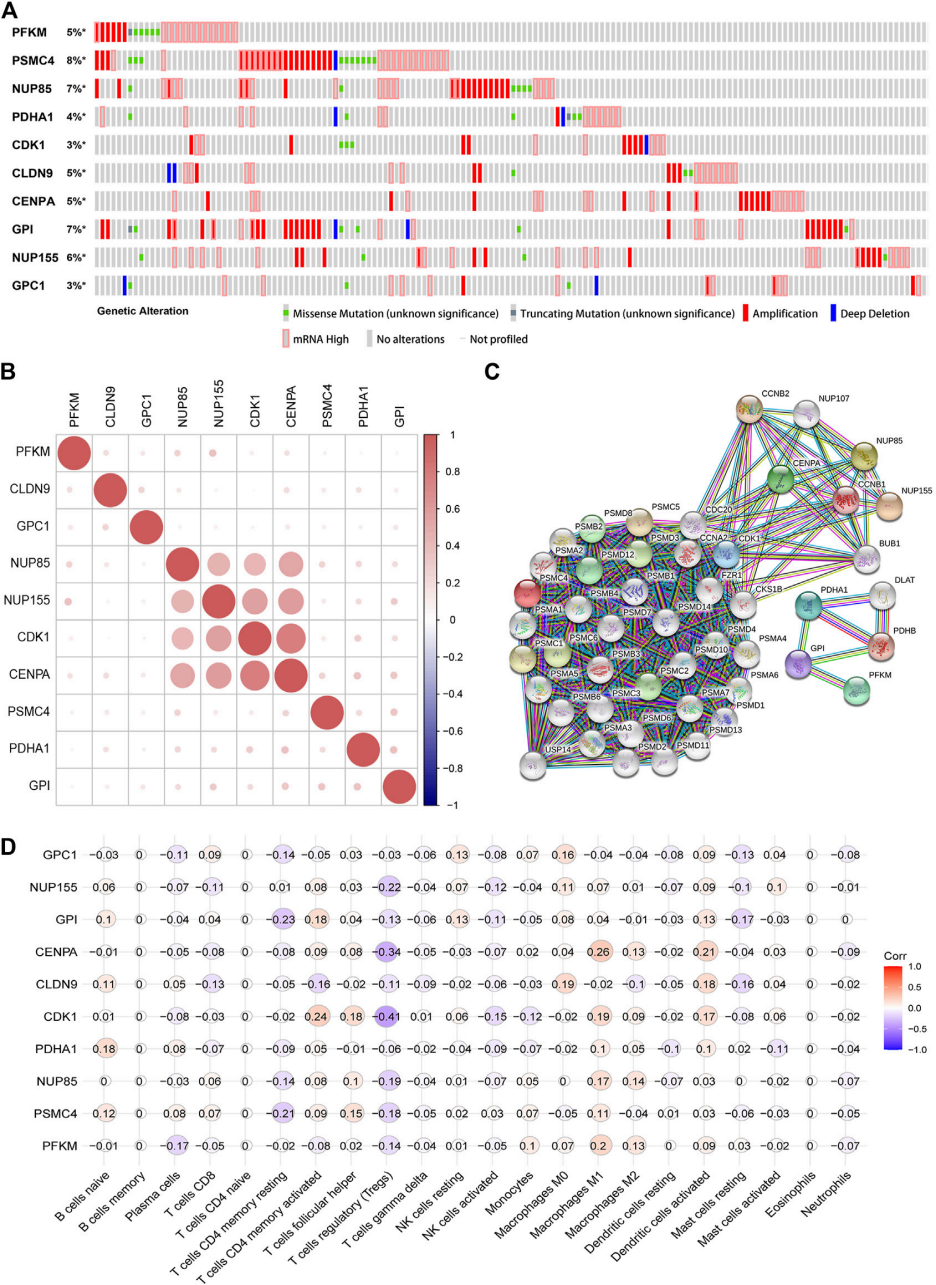
Comprehensive analysis of glycolysis related genes in the gene signature **(A)**. Gene alteration of selected genes in patients with EC from the cBioPortal website **(B)**. Co-expression analyses of 10 glycolysis-related gene signatures in EC by the R “corrplot” package **(C)**. Protein-protein interaction network of 10 gene signatures and other closely related proteins **(D)**. Correlation between the 10 glycolysis-related gene signatures and various immune cells. Red represents a positive correlation, and blue represents a negative correlation.

We analyzed the gene alteration of the 10 gene signature through the cBioPortal online website. The results showed that the expression alterations of PFKM, NUP85, PDHA1, CDK1, CLDN9, CENPA, GPI, NUP155, and GPC1 in endometrial carcinoma samples were 5, 7, 4, 3, 5, 5, 7, 6, and 3%, respectively. Amplification and increased mRNA were the most common changes (Figure 8A). Co-expression analysis showed that NUP85, NUP155, CDK1, and CENPA had a strong correlation (Figure 8B). Furthermore, we used the STRING database to analyze the proteins co-expressed and interacting with the 10 genes and constructed a PPI interaction network. Forty-eight proteins were screened in the PPI network (Figure 8C). The IntAct database was further used to construct an interaction network between the 10 genes and interactive genes (Supplementary Figure S3). In addition, we used immunohistochemistry results from the human protein atlas database to further verify the protein expression of the 10 genes in the prognostic signature. The results showed that the expression of PDHK1, NUP85, CDK1, CENPA, GPI, GPC1, PSMC4, and PFKM in EC was higher than that in normal endometrium, and NUP155 was not detected in EC and normal endometrium, although no data were found for CLDN9 (Supplementary Figure S4). In addition, we analyzed the relationship between the 10 genes and the clinical stage and found that the expression of PFKM, NUP85, PDHA1, CDK1, CLDN9, CENPA, GPI, NUP155, and GPC1 increased with increasing clinical stage (*p* < 0.05) (Supplementary Figure S5). Further, we analyzed the association between the 10 genes and immune cell infiltration. The results indicated that 10 genes were negatively correlated with T cell regulation (Tregs), including CDK1 (r = -0.41), CENPA (r = -0.34), and NUP155 (r = - 0.22) (Figure 8C). Taken together, the results might provide us with insights into function of the 10 glycolysis related genes in the progression of EC.

### Validation of the Glycolysis-Related Gene Signature Based on the Clinical Samples

The glycolysis-related gene signature was further validated by our own clinical data. A total of 24 EC samples from surgical patients in the Department of Obstetrics and Gynecology, Peking University People’s Hospital were used for the validation cohort. The EC patients in the validation cohort were divided into the low- and the high-risk subgroups according to the median risk score based on the above formula. K-M survival analysis showed that the overall survival rate of patients in the high-risk subgroup had obviously decreasing tendency compared with the low-risk subgroup, although the *p-*value is greater than 0.05 (*p* = 0.1044) (Supplementary Figure S6A). Further, the heatmap was used to show the expression difference of the 10 genes in the high- and low-risk subgroups. The results indicated that the expression of PFKM, CENPA, CDK1, GPI, NUP155, NUP85, and PDHA1 were significantly increased in the high-risk group compared with low-risk group, which were consistent with the results in the TCGA cohort (Supplementary Figure S6B). In addition, the expression of CDK1, CENPA, NUP155, and PSMC4 were found to be positively correlated with myometrial invasive (MI) (*p <* 0.05). The expression of CDK1, CENPA, NUP155, and PSMC4 in patients with deep-MI (Positive) were higher than that with superficial-MI (Negative) (Supplementary Figure S6C–F). It has been reported that the risk of lymph node metastasis was significantly increased in patients with EC and deep-MI (Singh et al., 2019). Thus, the high expression of these genes is closely related to the poor prognosis of EC patients.

### Inhibition of CDK1 Inhibits the Migration and Promotes the Apoptosis of EC cells

**FIGURE 9:**
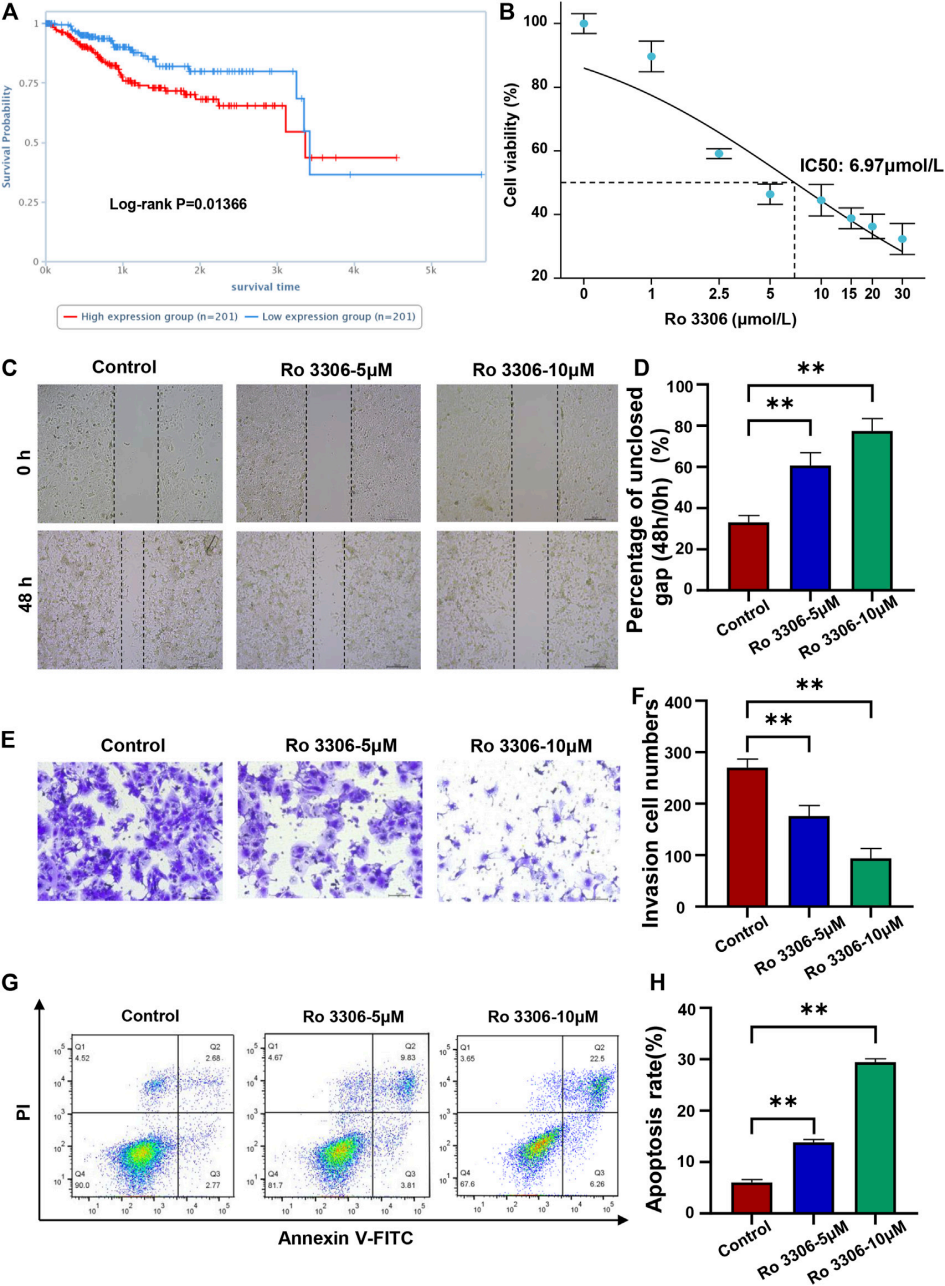
Inhibition of CDK1 inhibits the migration and promotes the apoptosis of EC cells **(A)**. High expression of CDK1 protein was closely related to the poor prognosis of EC patients (*p* < 0.05). The cancer proteome atlas (https://www.tcpaportal.org/tcpa/survival_analysis.html) **(B)**. The CCK-8 showed that the IC50 of Ro 3306 on Ishikawa cells was 6.97 μmol/L **(C,D)**. The scratch test revealed that compared with control group, cells treated with 5 μM or 10 μM Ro 3306 showed significantly increased gap size of oh **(E,F)**. The transwell experiment revealed that compared with control group, cells treated with 5 μM or 10 μM Ro 3306 showed significantly decreased invasion cell numbers **(G,H)**. Detection of apoptosis of Ishikawa cells treated with 5 μM or 10 μM Ro 3306 by flow cytometry. **p < 0.01, compared with control.

To further study the association between the 10 glycolysis related genes and survival of EC patients, the clinical prognosis analysis of proteins corresponding to the genes in the signature was performed on the cancer proteome atlas. We found that among the 10 proteins, high expression of CDK1 protein was closely related to the poor prognosis of EC patients (*p <* 0.05) (Figure 9A). Ro 3306 is an effective and selective CDK1 inhibitor. To verify the function of CDK1 in EC cells, we detected the effect of Ro 3306 on the proliferation, migration, and apoptosis of Ishikawa cells. The CCK-8 showed that the IC50 of Ro 3306 on Ishikawa cells was 6.97 μmol/L (Figure 9B). Then, we used 5 and 10 μM Ro 3306 for subsequent study. The scratch test and transwell experiment revealed that compared with the control group, cells treated with 5 μM or 10 μM Ro 3306 showed significantly decreased migration ability (Figures 9C–F). Also, Ishikawa cells treated with 5 μM or 10 μM Ro 3306 caused more apoptosis than the control group in a concentration-dependent manner (Figures 9G,H). To further explore the mechanism of CDK1, a protein regulatory network of CDK1 was constructed based on the BioGRID database, some proteins in the PPI network were consistent with the STRING database (Supplementary Figure S7). These results suggest that the CDK1 protein may be a key factor affecting the prognosis of EC and a potential therapeutic target for EC.

## Discussion

In recent years, the role of metabolic reprogramming in tumors has been widely studied. Glycometabolism reprogramming is one of the characteristics that tumor cells are different from normal cells. Even under the condition of sufficient oxygen, tumor cells are more likely to use glycolysis for rapid energy supply. Therefore, studying the relationship between metabolic reprogramming and tumor development is becoming a new method for tumor diagnosis, prevention, and treatment. At present, many studies have reported the relationship between glycolysis and EC (Shim et al., 2014; Han et al., 2019). However, research on biomarkers related to glycolysis in EC remains limited. It has been reported that clinical characteristics, such as age, stage, grade, and lymph node metastasis, cannot accurately predict the prognosis of patients (Zhao et al., 2019). As a result, an increasing number of studies are exploring gene biomarkers, and many studies have found that developing multiple gene-related risk models can improve the prediction efficiency (Zeng et al., 2019; Tao et al., 2020). Therefore, the purpose of this study was to explore the glycolysis-related prognostic biomarkers of EC and further to analyze their relationship with immune cell infiltration.

We first downloaded glycolysis-related gene sets from GSEA and screened DEGs between EC and normal samples, including 128 upregulated genes and 28 downregulated genes. Furthermore, we used GO and KEGG enrichment analysis to verify the biological function and signaling pathways of DEGs. Next, we used univariate Cox regression to initially screen genes related to the prognosis of EC and further used LASSO regression analysis to screen and construct the prognostic gene signature. A total of 10 mRNAs (PFKM, PSMC4, NUP85, PDHA1, CDK1, CLDN9, CENPA, GPI, NUP155, and GPC1) significantly related to the overall survival of EC were identified to construct the prognostic gene signature, which was identified as an independent prognostic factor for EC. Furthermore, ROC curve analysis was conducted to verify the prognostic value of the gene signature, showed that the area under the ROC curve of the risk score was greater than that of stage, grade, age, and LNM. It indicates that the predictive value of this gene signature is better than traditional prognostic indicators. The glycolysis-related gene signature was further validated by our own clinical data including 24 EC samples. K-M survival analysis showed that the overall survival rate of patients in the high-risk subgroup had obviously decreasing tendency compared with the low-risk subgroup in the validation cohort, although the *p*-value is greater than 0.05 (*p* = 0.1044), maybe it is because of smaller sample size, different populations, and sequencing batches compared to the TCGA database. Nonetheless, the expression of PFKM, CENPA, CDK1, GPI, NUP155, NUP85, and PDHA1 was significantly increased in the high-risk group compared with the low-risk group, which was consistent with the results in the TCGA cohort. In addition, we integrated multiple prognostic factors (including risk score, stage, grade, and age) to construct a nomogram to effectively predict the 1–5 years survival rate of patients, which may help to plan short-term follow-up of individualized treatment.

In the past, many studies have focused on the role of glycolysis in tumors. It has been reported that aerobic glycolysis in tumors constantly produces lactic acid, which provides energy for the tumor, and the increased lactic acid in the microenvironment could also affect the immunotherapy effect (Bohn et al., 2018). It has also been found that antitumor metabolism therapy combined with immunotherapy can effectively inhibit tumor growth (Gao et al., 2019). To further explore the relationship between the glycolysis-related gene signature and immune cell infiltration and immune function, we first analyzed the immune scores, stromal scores, and ESTIMATE scores of patients in the high-risk subgroup and low-risk subgroup based on the 10 gene signature. We found that the immune scores, stromal scores, and ESTIMATE scores of patients in the high-risk subgroup were significantly lower than those of the low-risk subgroup. At the same time, the overall survival rate of patients with low immune scores and estimated scores was significantly worse than that of patients with high scores. Some studies have shown that the more immune cells enter the tumor metastasis, the higher the immune score is, the higher the survival rate is, and the lower the recurrence rate is. Metastasis with the smallest number of immune cells entering represented the worst immune microenvironment, and immune escape was most likely to occur under this condition (Van den Eynde et al., 2018). These results suggested that the poor prognosis of patients in the high-risk subgroup might be closely related to the low immune scores. However, whether the activation of glycolysis-related pathways affects the infiltration of immune cells warrants further investigation.

We further found that many immune cells, such as activated dendritic cells, M1 macrophages, M2 macrophages, memory activated T cells, and follicular helper T cells, were significantly higher in the high-risk subgroup than in the low-risk subgroup, while dendritic cell resting, memory resting T cells, and regulatory T cells (Tregs) were significantly lower in the high-risk subgroup. There was a positive correlation between the three immune cells and the overall survival rate of patients, including dendritic cell resetting, NK cells activated, and T cell regulation (Treg). According to previous research, resting dendritic cells exist in most tissues and are activated to mature antigen-presenting cells under external stimulation. Antigen presentation by resting dendritic cells could induce protective immunity (Probst et al., 2005). Tregs play a key role in maintaining immune system homeostasis. Some studies have shown that the high density of Treg cells in tumors is related to the clinical prognosis of tumors, such as liver cancer and gastric cancer (Najafi et al., 2019). High proportions of Tregs among tumor-infiltrating CD4^+^ T cells were favorable (Choi et al., 2016). It is suggested that the poor prognosis of patients in the high-risk group may be related to the imbalance of immune cells in the tumor, especially the decrease of dendritic cell resting, NK cells activated, and Treg cells.

To further study the potential molecular mechanism of prognostic gene signature in EC, WGCNA and GSEA were performed. The WGCNA results indicated that the 10 gene signature was significantly associated with a functional gene module that was mainly enriched in cell division, regulation of cell cycle process, and DNA replication. It is noteworthy that there was significant correlation between the gene module and clinical grade of patients. GSEA enrichment analysis revealed that many signaling pathways were significantly enriched in the high-risk subgroup, including pathways related to metabolism and metabolic diseases, such as pyruvate metabolism, glycolysis gluconeogenesis, and insulin signaling pathways. It has been reported that both malignant transformation and tumor development, including invasion and metastasis, required metabolic reprogramming (DeBerardinis et al., 2008). Metabolic heterogeneity is an important reason for the failure of treatment to produce the same effect on cancer cells (Yoshida, 2015). High insulin level is an independent risk factor of EC. Increased insulin and IGF-1 could activate downstream signaling pathways by binding with IR and IGF-1 receptor to promote the proliferation of EC cells (Mu et al., 2012). These results suggested that the poor prognosis of patients in the high-risk subgroup might be closely related to tumor metabolic reprogramming and the activation of metabolic disease-related pathways. In addition, other pathways closely related to tumorigenesis and development were also significantly enriched in the high-risk subgroup, such as the cell cycle, EC, the ERBB signaling pathway, the MAPK signaling pathway, the mTOR signaling pathway, and the Wnt signaling pathway. Taken together, these results show that the 10 gene signature is closely related to metabolic imbalance and provide a potential molecular mechanism for elucidating the relationship between the gene signature and EC progression.

In the 10 gene signature, most genes have been reported to be closely related to the occurrence and development of cancer. PFKM, the second rate-limiting enzyme in the glycolysis pathway, has been shown to be closely related to the increased risk of breast cancer (Ahsan et al., 2014). PSMC4 is a member of the proteasome complex, which is responsible for recognizing ubiquitin-labeled substrates and ingesting them into the proteasome (19S regulatory complex). The overexpression of PSMC4 promoted the degradation of some key cell regulatory proteins, such as tumor suppressors, and further promoted the progression of tumors (Hellwinkel et al., 2011). Therefore, inhibition of the proteasome is a promising cancer treatment strategy. The nucleoporins NUP155 and NUP85 were reported to be upregulated in hepatocellular carcinoma, accompanied by TP53 silencing and overexpression of cell cycle-related genes (Beck et al., 2017). PDHA1 is the main regulatory site of PDH activity. PDHA1 regulates the deactivation or activation of PDH through phosphorylation and dephosphorylation and then affects the mitochondrial tricarboxylic acid cycle and glycolysis metabolic flow. It has been reported that the expression of PDHA1 is abnormal in a variety of tumors, and it is closely related to tumor invasion, drug resistance, and prognosis by affecting tumor cell glucose metabolism. The upregulation of PDHA1 could promote the metastasis of cholangiocarcinoma (Dan Li et al., 2018). In contrast, another study reported that low expression of PDHA1 predicted poor prognosis in gastric cancer (Song et al., 2019). Overexpression of CLDN9 could promote tumor cell invasion through Tyk2/STAT3 signaling (Liu et al., 2019a). Upregulation of GPI-anchored proteins could promote tumor cell migration and progression by enhancing the ERBB signaling pathway (Zhang et al., 2019). GPC1 has received growing interest in recent years due to its high capability of visualizing soft tissue, and GPC1 has been reported to have potential value in the diagnosis of breast cancer (Li et al., 2019). CENPA and CDK1 were also identified as prognostic markers of lung cancer (Liu et al., 2018). In our study we found that the expression of PFKM, NUP85, PDHA1, CDK1, CLDN9, CENPA, GPI, NUP155, and GPC1 increased with increasing clinical stage, exhibiting their role in tumor progression. A PPI network containing 48 proteins was constructed to show the correlation between the 10 genes and potential interacting proteins. IntAct database was also used to show the physical association and direct interaction between the 10 genes and related proteins. There was a direct interaction between PDHA1 and PDHB; PSMC4 and PSMC5; CDK1 and CCNB1 both in the PPI network from the STRING database and the IntAct database. We also found CDK1 protein was closely related to the poor prognosis of EC patients, and the expression of CDK1 was negatively correlated with Treg cells infiltration. Thus, CDK1 may be involved in regulating the infiltration of Treg cells. Also, inhibition of CDK1 has been proved to inhibit the migration and promote the apoptosis of EC cells. Since the BioGRID database has certain advantages in predicting interacting proteins for single gene or protein (Oughtred et al., 2019), a protein regulatory network of CDK1 was constructed based on the BioGRID database. It showed that some proteins such as BUB1 and CCNB1 in the network were consistent with the PPI network from the STRING database. BUB1 and CCNB1 have been reported to have important prognostic value in EC (Li et al., 2013; Liu et al., 2019b). Taken together, these results indicate that CDK1 may be used as a therapeutic target for EC patients.

## Conclusion

In this study, we identified a glycolysis-related 10 gene signature for predicting the prognosis of EC patients based on TCGA, where higher risk scores represent a worse prognosis. The gene signature was identified as an independent prognostic factor for EC and has been tested to have good survival predictive ability. A nomogram based on the gene signature and other clinical prognostic factors was constructed to effectively predict the 1–5 years survival rate of EC patients. In addition, the poor prognosis of patients in the high-risk subgroup might be closely related to the low immune scores and the imbalance of immune cells in tumor. Finally, CDK1 was identified to be a potential prognostic biomarker and therapeutic target for EC patients.

## Data Availability

The datasets presented in this study can be found in online repositories. The names of the repository/repositories and accession number(s) can be found in the article/Supplementary Material. Further inquiries can be directed to the corresponding author.
