# Supplementary material for: Comprehensive Analysis of the Glycolysis-Related Gene Prognostic Signature and Immune Infiltration in Endometrial Cancer
Source: Front Cell Dev Biol. 2022 Feb 11;9:797826. doi: 10.3389/fcell.2021.797826 (PMC8879138; doi:10.3389/fcell.2021.797826)
Supplement: Supplementary file 1 [file DataSheet1.docx]

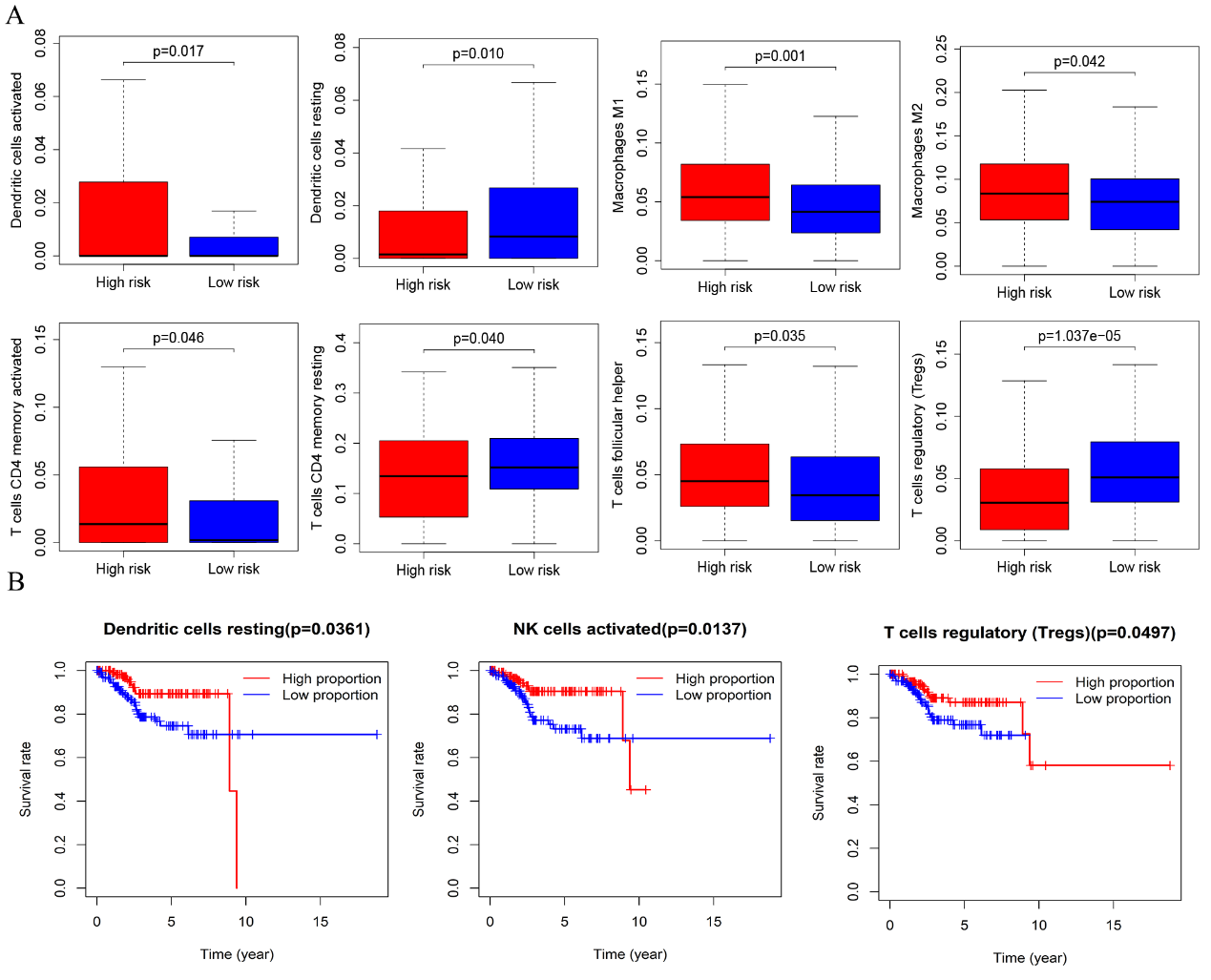


**Figure S1**. A. Abundance ratios of the immune cells for patients in the low- vs. high-risk group. B. Kaplan-Meier analysis of the relationship between the infiltration of immunocytes and overall survival for patients (filter criteria: *P*<0.05).


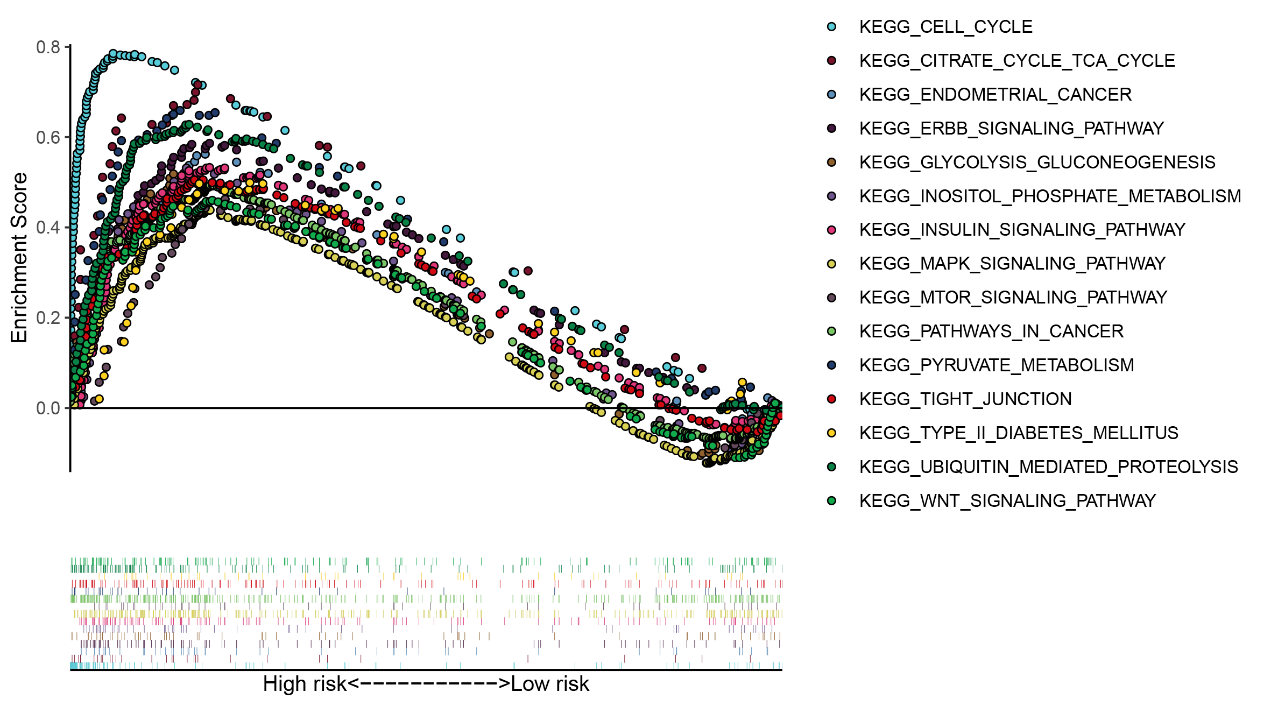


**Figure S2**. The Fifteen representative significantly enriched KEGG pathways in high-risk by GSEA.


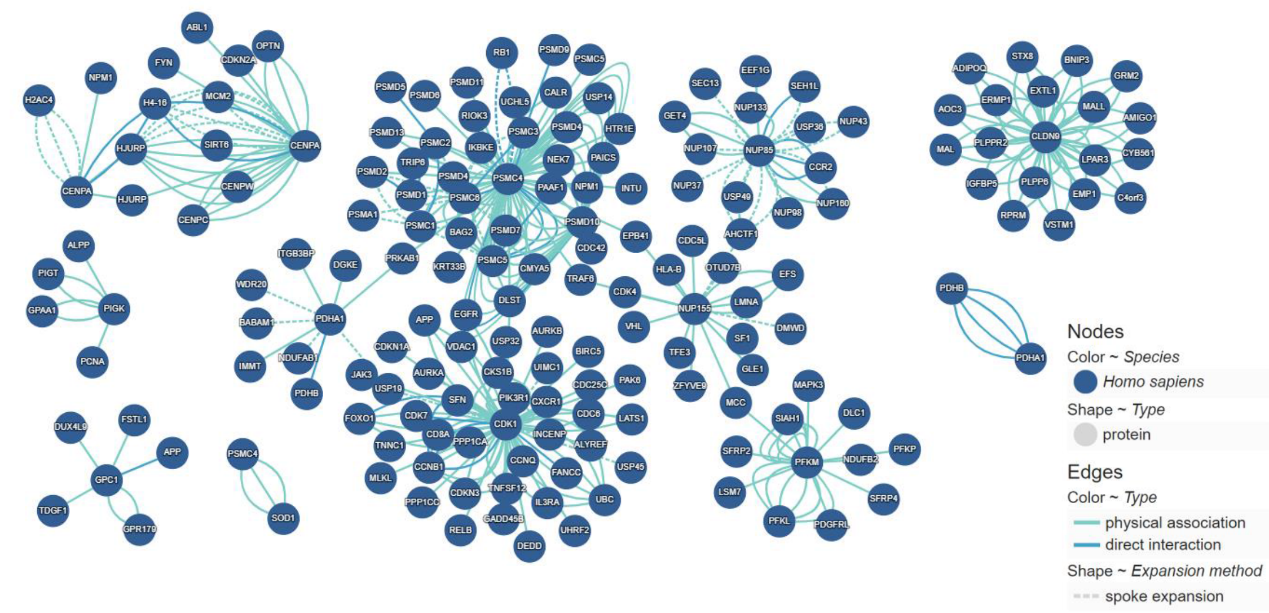


Figure S3 The IntAct database was used to construct a interaction network between the ten genes in the gene signature and interactive genes.


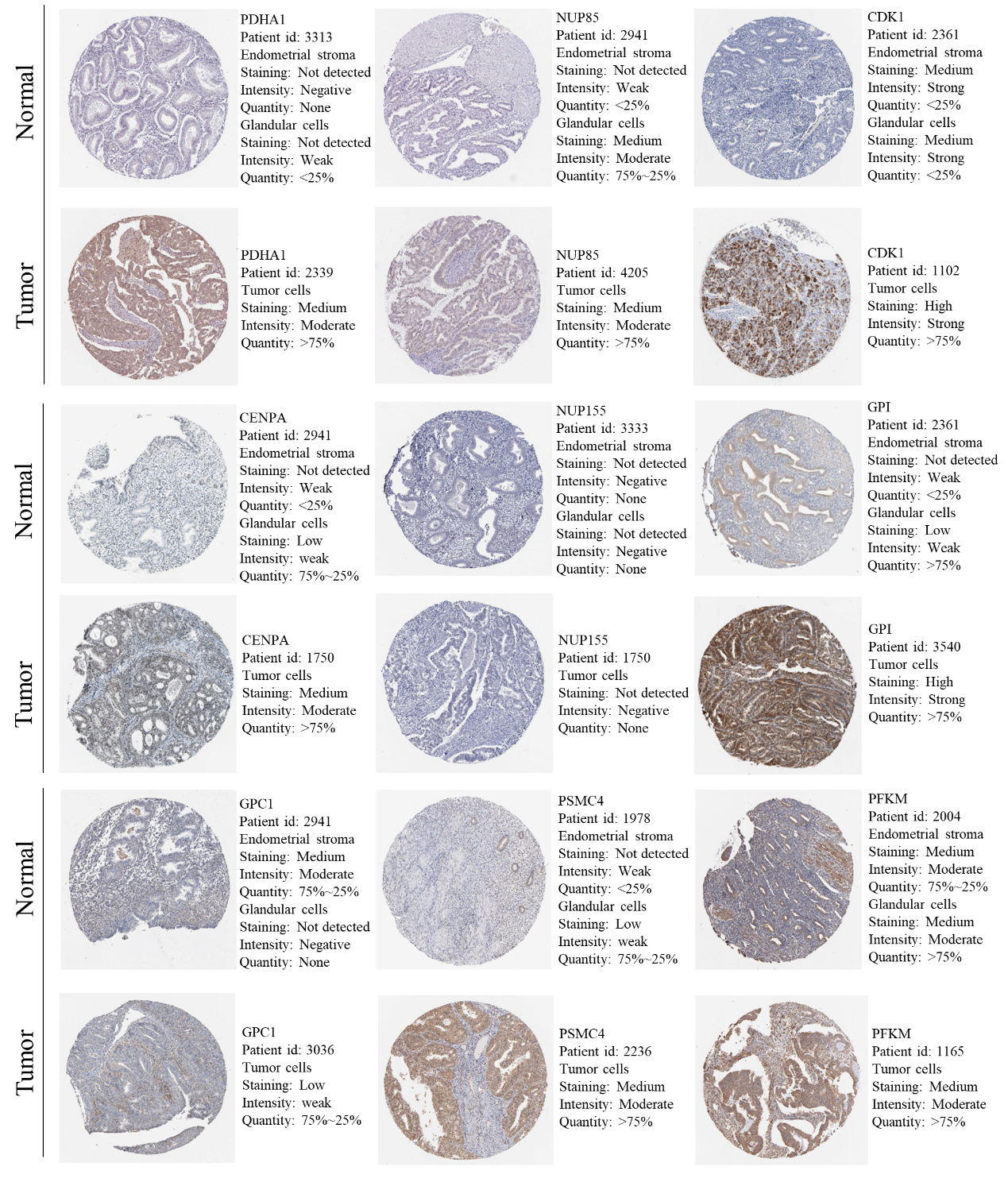


**Figure S4.** The representative protein expression of ten glycolysis-related gene signature in EC and normal tissue. Data were from the Human Protein Atlas (<http://www>. Proteinatlas.org) database. There was no data of CLDN9 in the database.


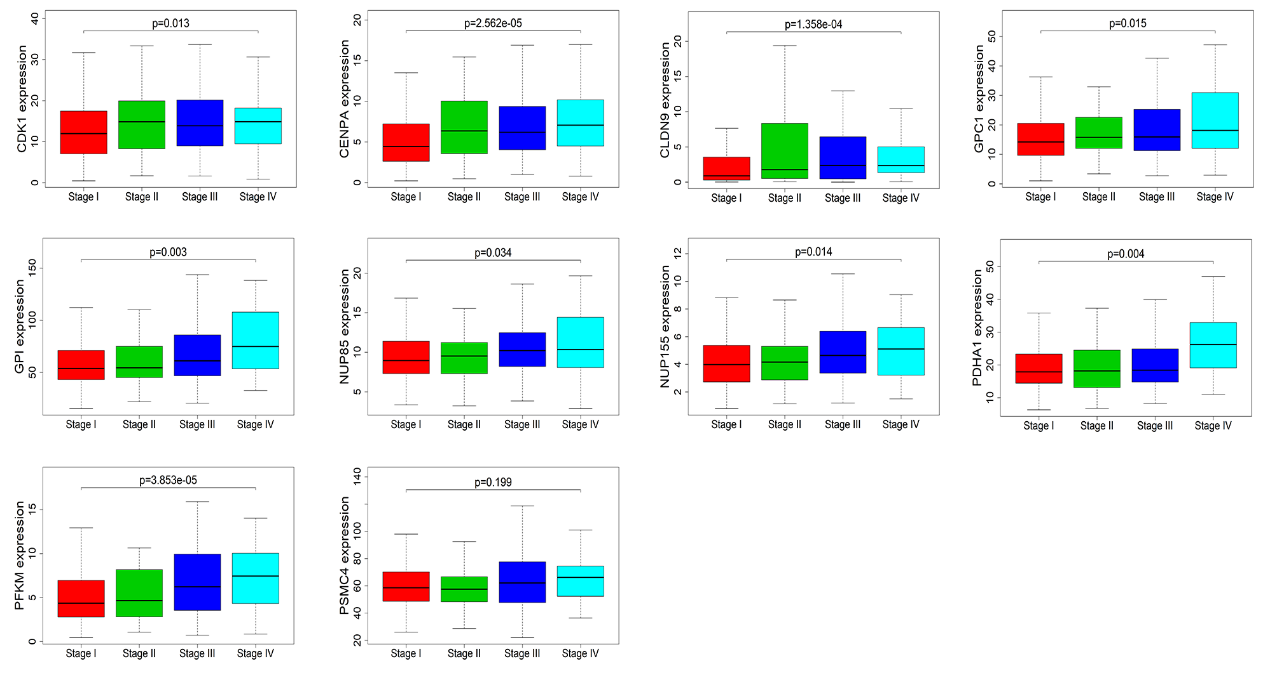


**Figure S5**. The correlation between ten glycolysis-related gene signature and clinical stages. The expression of PFKM、NUP85、PDHA1、CDK1、CLDN9、CENPA、GPI、NUP155 and GPCI increased with the increase of clinical stage (*P* < 0.05) .


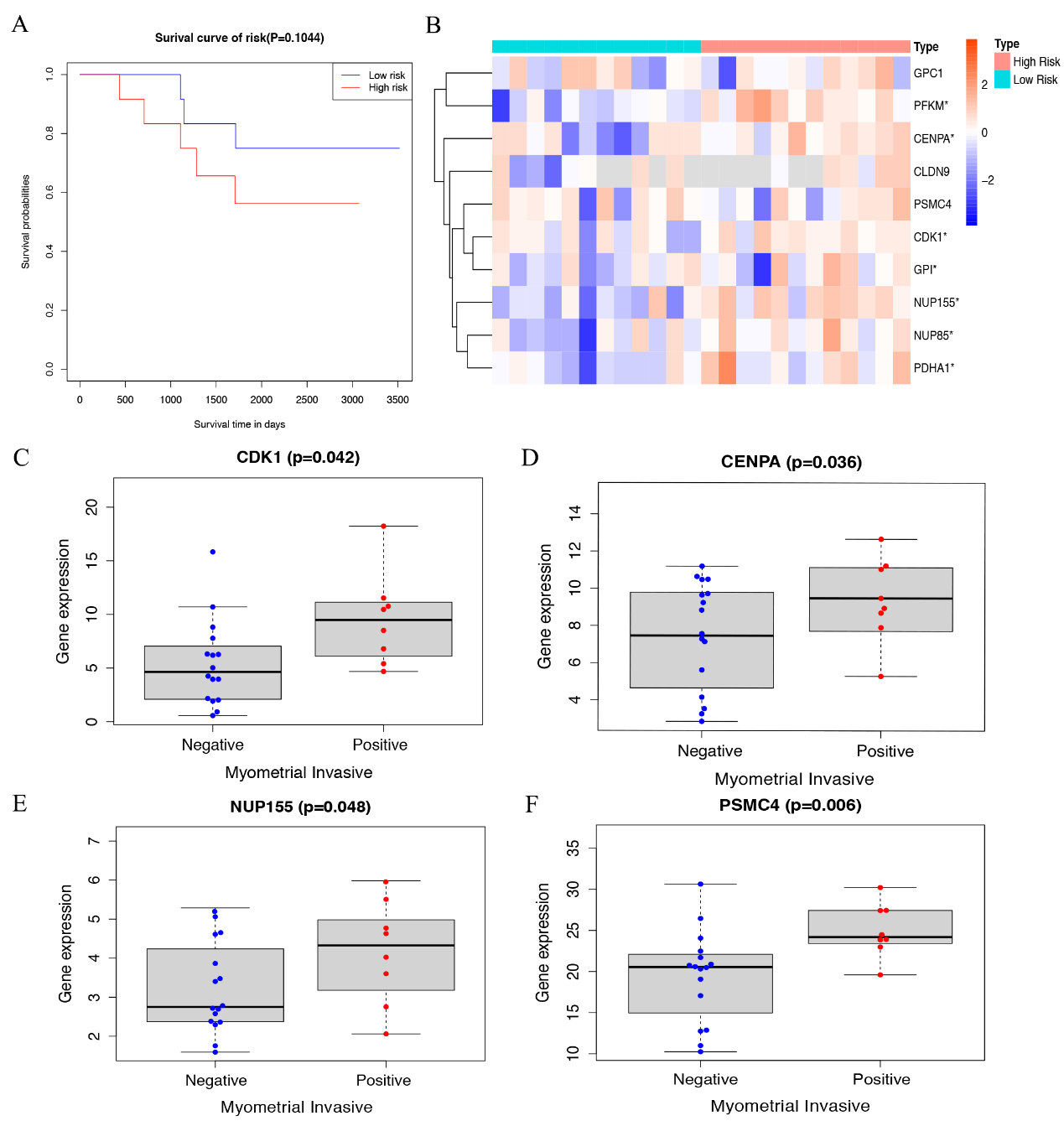


**Figure S6**. Validation of the glycolysis-related gene signature based on the clinical samples. A. K-M survival analysis of overall survival for patients with low and high-risk. B. The heatmap of the ten glycolysis-related genes in low and high-risk subgroup.

C-F. The association between the expression of CDK1, CENPA, NUP155, PSMC4 and mynometrial invasion of EC patients.


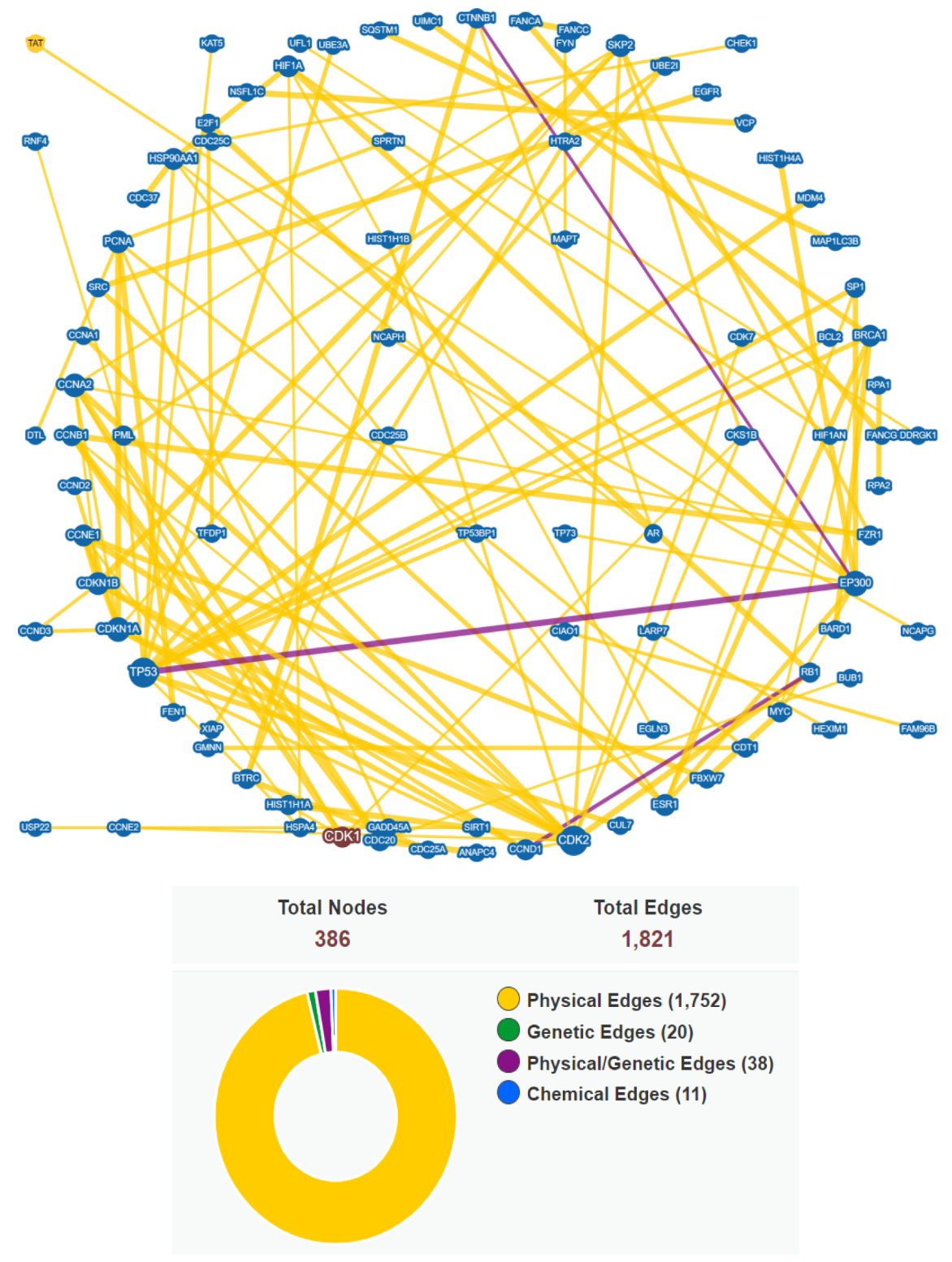


**Figure S7.** A protein regulatory network of CDK1 was constructed based on the BioGRID database.

**Table S1.** The result of LASSO analysis in EC.

| **Gene** | **Coef** |
| --- | --- |
| PFKM | 0.0420 |
| PSMC4 | 0.0032 |
| NUP85 | 0.0097 |
| PDHA1 | 0.0138 |
| CDK1 | 0.0028 |
| CLDN9 | 0.0024 |
| CENPA | 0.0252 |
| GPI | 0.0004 |
| NUP155 | 0.0253 |
| GPC1 | 0.0067 |
